# Perceived appropriateness of alcohol screening and brief advice programmes in Colombia, Mexico and Peru and barriers to their implementation in primary health care – a cross-sectional survey

**DOI:** 10.1017/S1463423620000675

**Published:** 2021-01-28

**Authors:** Daša Kokole, Liesbeth Mercken, Eva Jané-Llopis, Guillermina Natera Rey, Miriam Arroyo, Perla Medina, Augusto Pérez-Gómez, Juliana Mejía-Trujillo, Marina Piazza, Ines V. Bustamante, Amy O’Donnell, Eileen Kaner, Antoni Gual, Hugo Lopez-Pelayo, Bernd Schulte, Jakob Manthey, Jürgen Rehm, Peter Anderson, Hein de Vries

**Affiliations:** 1 Department of Health Promotion, CAPHRI Care and Public Health Research Institute, Maastricht University, 6200 MD, Maastricht, The Netherlands; 2 University Ramon Llull, ESADE, Barcelona, Spain; 3 Institute for Mental Health Policy Research, CAMH, Toronto, ON M5S 2S1, Canada; 4 Instituto Nacional de Psiquiatría Ramón de la Fuente Muñiz, Calz México-Xochimilco 101, Huipulco, 14370 Ciudad de México, Mexico; 5 Corporación Nuevos Rumbos, Bogotá, Colombia; 6 School of Public Health and Administration, Universidad Peruana Cayetano Heredia, San Martin de Porres, Peru; 7 Population Health Sciences Institute, Newcastle University, Newcastle upon Tyne NE2 4AX, UK; 8 Red de Trastornos Adictivos, Instituto Carlos III, Sinesio Delgado, 4, 28029 Madrid, Spain; 9 Addictions Unit, Psychiatry Department Hospital Clínic, Villarroel 170, 08036 Barcelona, Spain; 10 Institut d’Investigacions Biomèdiques August Pi Sunyer (IDIBAPS), Rosselló, 08036 Barcelona, Spain; 11 Center for Interdisciplinary Addiction Research (ZIS), Department of Psychiatry and Psychotherapy, University Medical Center Hamburg-Eppendorf, Hamburg, Germany; 12 Institute for Clinical Psychology and Psychotherapy, TU Dresden, Chemnitzer Str. 46, 01187 Dresden, Germany; 13 Dalla Lana School of Public Health, University of Toronto, Toronto, ON M5T 3M7, Canada; 14 Department of Psychiatry, University of Toronto, Toronto, ON M5T 1R8, Canada; 15 Department of International Health Projects, Institute for Leadership and Health Management, I.M. Sechenov First Moscow State Medical University, 119992 Moscow, Russian Federation

**Keywords:** alcohol screening and brief advice, appropriateness, barriers, implementation, middle-income country

## Abstract

**Background:**

Providing alcohol screening and brief advice (SBA) in primary health care (PHC) can be an effective measure to reduce alcohol consumption. To aid successful implementation in an upper middle-income country context, this study investigates the perceived appropriateness of the programme and the perceived barriers to its implementation in PHC settings in three Latin American countries: Colombia, Mexico and Peru, as part of larger implementation study (SCALA).

**Methods:**

An online survey based on the Tailored Implementation for Chronic Diseases (TICD) implementation framework was disseminated in the three countries to key stakeholders with experience in the topic and/or setting (both health professionals and other roles, for example regional health administrators and national experts). In total, 55 respondents participated (66% response rate). For responses to both appropriateness and barriers questions, frequencies were computed, and country comparisons were made using Chi square and Kruskal–Wallis non-parametric tests.

**Results:**

Alcohol SBA was seen as an appropriate programme to reduce heavy alcohol use in PHC and a range of providers were considered suitable for its delivery, such as general practitioners, nurses, psychologists and social workers. Contextual factors such as patients’ normalised perception of their heavy drinking, lack of on-going support for providers, difficulty of accessing referral services and lenient alcohol control laws were the highest rated barriers. Country differences were found for two barriers: Peruvian respondents rated SBA guidelines as less clear than Mexican (Mann–Whitney *U* = −18.10, *P* = 0.001), and more strongly indicated lack of available screening instruments than Colombian (Mann–Whitney *U* = −12.82, *P* = 0.035) and Mexican respondents (Mann–Whitney *U* = −13.56, *P* = 0.018).

**Conclusions:**

The study shows the need to address contextual factors for successful implementation of SBA in practice. General congruence between the countries suggests that similar approaches can be used to encourage widespread implementation of SBA in all three studied countries, with minor tailoring based on the few country-specific barriers.

## Introduction

In all global comparative risk assessments, alcohol use is amongst the 10 leading risk factors for both deaths and disability adjusted life years (Rehm and Imtiaz, 2016; GBD 2016 Alcohol Collaborators, 2018) and has been estimated to cause about 3 million deaths annually (Shield *et al.*, 2020). It has been linked with increasing the risk of a number of diseases including alcohol use disorders (Grant *et al.*, 2015), cancers (Bagnardi *et al.*, 2015), liver disease (Rehm *et al.*, 2010), infectious diseases (Imtiaz *et al.*, 2017) and ischaemic (for heavy drinking occasions)(Roerecke and Rehm, 2014) as well as non-ischaemic cardiovascular disease (Rehm and Roerecke, 2017). Although the highest levels of per capita alcohol consumption are found in the European region (World Health Organisation, 2018), the pattern of high levels of alcohol consumption is also prevalent in the Latin American region (Manthey *et al.*, 2019), along with a high level of negative consequences (World Health Organisation, 2018). In Colombia, Mexico, and Peru, the three Latin American countries included in this study, alcohol use ranked as the fifth (in Mexico) and sixth (in Colombia and Peru) highest risk factor for death and disability in 2017 (Institute for Health Metrics and Evaluation, 2019a, 2019b, 2019c). The estimated percentages of deaths attributable to alcohol in the three countries ranged between 6.4 and 11% for males and 1.2–2.1% for females, and percentages of total attributable disability adjusted life years were above the world average at 7.6–12% for males and 2.1–3% for females (Gakidou *et al.*, 2017; GBD 2016 Alcohol Collaborators, 2018). These estimations show that the three countries could benefit from widespread implementation of measures to decrease heavy drinking in order to reduce the alcohol-related harm.

There is a large and robust evidence base demonstrating positive impacts for alcohol screening and brief advice (SBA) programmes, particularly when delivered in primary health care (PHC) settings. Over 70 randomised controlled trials suggest these simple interventions are both clinically and cost-effective at helping clinicians to identify patients drinking excessively and to provide short, structured advice to those needing to reduce their alcohol consumption (O’Donnell *et al.*, 2014; Kaner *et al.*, 2018). While evidence for the effectiveness of alcohol SBA in PHC comes mainly from studies in high-income countries (HIC) (O’Donnell *et al.*, 2014), emerging evidence points to its effectiveness also in middle-income countries (MIC) (Joseph and Basu, 2017), including in the Latin American region (Ronzani *et al.*, 2009; Moretti-Pires and Corradi-Webster, 2011). Evidence from PHC settings in HIC also shows that despite the established effectiveness of alcohol SBA, uptake in routine care remains low (Colom *et al.*, 2014; O’Donnell *et al.*, 2014). Likewise, although there are on-going efforts to introduce SBA in Latin American countries (Gelberg *et al.*, 2017), widespread implementation has still not been achieved.

Scaling up SBA programmes will increase the number of patients detected to drink excessively and receiving advice on how to cut down, which could in turn lead to reduced alcohol consumption among the identified risky drinkers and its associated individual and wider societal harms. When aiming to scale up alcohol SBA in a new context however, it is beneficial to engage and consult with local stakeholders in order to adapt the intervention and increase the likelihood of successful and widespread implementation (Theobald *et al.*, 2018). This study assessed the perspectives of key local stakeholders in three municipalities in Colombia, Mexico and Peru on two aspects relevant for successful implementation of SBA in practice: perceived appropriateness of the intervention and barriers to adoption.

First, appropriateness has been defined as the perceived fit, relevance or compatibility of the evidence-based programme for a given practice setting, provider or consumer and/or the perceived fit of the intervention to address a particular issue or problem (Proctor *et al.*, 2011). Assessment of appropriateness can provide an insight to the social validity of the intervention as perceived in the local context (World Health Organisation, 2016) and to help understand the implementation processes once the intervention is implemented (Proctor *et al.*, 2011). There is currently a lack of information on perceived appropriateness of alcohol SBA in PHC settings in the Latin American context, and no other studies assessing this issue have been identified in the literature.

Second, studying existing or potential barriers to delivery can help identify the reasons behind the evidence-practice gap for a specific intervention or initiative, and thus support the development of more effective strategies to improve successful implementation (World Health Organisation, 2016). A large body of literature on barriers to alcohol SBA in PHC exists, suggesting lack of time, lack of training, providers’ attitudes and lack of organisational support, as core factors affecting delivery (Johnson *et al.*, 2011; Rahm *et al.*, 2015; Abidi *et al.*, 2016; Derges *et al.*, 2017; Vendetti *et al.*, 2017), However, most of this evidence comes from HIC (eg, UK, US, Finland, Sweden, Australia) (Johnson *et al.*, 2011; Derges *et al.*, 2017), and there is less knowledge of whether the barriers are the same in low- and middle-income countries (LMIC). In Latin America, for example, the few published studies have focussed on barriers to SBA implementation in specialised rather than PHC settings (Hoffman *et al.*, 2016; Isela *et al.*, 2016), and identified factors such as lack of standardised guidelines, lack of training of the providers, lack of providers’ perceived role responsibility, lack of time, lack of proper infrastructure and diversity of users affecting their delivery. These barriers echo some of those found in HIC (Johnson *et al.*, 2011; O’Donnell *et al.*, 2014; Derges *et al.*, 2017). However, the evidence suggests there are also some region-specific barriers, such as the lack of proper facilities to deliver the intervention.

In order to facilitate the assessment and comparison of barriers between countries, the Tailored Implementation for Chronic Diseases (TICD) framework was used (Flottorp *et al.*, 2013). This framework groups the determinants of practice into seven domains: guideline factors, individual health professional factors, patient factors, professional interactions, incentives and resources, capacity for organisational change and social, political and legal factors (Flottorp *et al.*, 2013). The latter five domains can be further framed as contextual factors (Nilsen and Bernhardsson, 2019). The added value of using such a framework is the recognition of different levels of influence on practice, including the importance of context, going beyond the individual-level factors which are often overly prominent in alcohol SBA implementation studies (Vendetti *et al.*, 2017).

The main aim of the study was thus twofold. First, the study aimed to assess and compare the perceived overall appropriateness of the alcohol screening and brief advice from the perspective of local stakeholders in three municipalities in Colombia, Mexico and Peru. Second, the study aimed to assess and compare the key stakeholders’ perspective on the barriers to implementation of SBA in the three countries and explore any differences based on their occupations.

## Methods

### Design and setting

The study was carried out as part of a larger research project testing implementation strategies for SBA implementation in Colombia, Mexico and Peru (SCALA – Scale up of Prevention and Management of Alcohol Use Disorders and Comorbid Depression in Latin America) (Jane-LLopis *et al.*, 2020). A cross-sectional survey was disseminated in municipalities in the cities of Bogota, Lima and Mexico City. In order to maximise feasibility, the local researchers selected the municipalities based on their location in the country and existing networks. To further characterise the setting, main demographic and health care system characteristics of the three countries are presented in Table 1.

**Table 1.** Demographic and health system characteristics in Colombia, México and Perú

	Colombia	México	Perú
*Main country demographics*	In 2018, Colombia had population of 48 258 494. In total, 51.2% were female, 75.5% were living in urban areas. Age distribution was 24.0% under 15, 67% 15–64, 8.8% 65+.^1^	In 2015, Mexico had population of 119 938 473. In total, 51.4% were female, 76.8% were living in urban areas. Age distribution in 2010 was 29.3% under 15, 64.4% 15–64, 6.3% 65+.^2^	In 2017, Peru had population of 31 237 385. In total, 50.5% were female, 81.9% were living in urban areas. Age distribution was 26.5% under 15, 65.3% 15–64, 8.2% 65+.^3^
*Health care system, including PHC*	*Sistema General de Seguridad Social en Salud* (SGSSS, General System of Social Security in Health). Most people are affiliated with the SGSSS through contributory regime (employed people) or the subsidised regime (low income population, indigenous, displaced, incarcerated population). There is also the special benefit regime (armed forces, teachers, and a state-owned petroleum company) and private insurance (voluntary).^4^	Mexican health care works by three-tier system: IMSS (Mexican Social Security Institute) covers employees in private and public sector. Seguro Popular (recently replaced by Instituto Nacional Salud para el Bienestar) is set up for those who don’t qualify for IMSS tier due to financial reasons or because of preexisting conditions. There is also option of private insurance.^7^	The Peruvian health care system is a four-tier system, including the following: public (Ministry of Health and district facilities, police and armed forces facilities); the social insurance system (EsSalud) and private for-profit and private not-for-profit (nongovernmental organisation and religious) facilities. It is a decentralised health system, where the national level that sets overall policies and frameworks, and the regional and local authorities are responsible for implementation.^8^
	In 2016, the new Comprehensive Health Care Model (Modelo Integral de Atención en Salud, MIAS) was introduced, with the aim to strengthen primary health care delivery and improve population access to health care, through increasing the responsibility and decision-making capacity of health teams. ^9^	In 2015, a Comprehensive Health Care model (MAI) was introduced in order to standardise health care services, optimise health resources and infrastructure, and promote citizens’ participation, which placed PHC one of the most important strategies for health care in Mexico.^7^	There are three categories of facilities that provide PHC: primary (I-1 to I-4), secondary (II-1 and II-2) and tertiary facilities. PHC is provided through a doctor-supported infrastructure; only in category I-1 facilities are supported by nurses, midwives or health technicians.^8^
	In 2016, health insurance coverage reached 96% of the population, 26% lacked access to health services (data from 2016).^5^ Based on 2017 data, health expenditure represented 7% of GDP, out-of-pocket payments counted as 16% of current health expenditure^6^	In 2014, health insurance coverage reached 80% of the population, 20% lacked access to health services.^5^ Based on 2017 data, health expenditure represented 6% of GDP, out-of-pocket payments counted as 41% of current health expenditure. PHC Expenditure represented 44% of health expenditure.^6^	In 2016, health insurance coverage reached 76% of population, 66% lacked access to health services.^5^ Based on 2017 data, health expenditure re presented 5% of GDP, out-of-pocket payments counted as 28 % of current health expenditure.^6^
*Distribution of health professionals*	In 2018, there were 108 499 medical doctors (21.85 per 10 000 population) and 66 095 nursing and midwifery personnel (13.31 per 10 000 population).^10^	In 2017, there were 297 307 medical doctors (23.83 per 10 000 population) and 302 363 nursing and midwifery personnel (23.96 per 10 000 population).^10^	In 2016, there were 40 352 medical doctors (13.05 per 10 000 population) and 78 048 nursing and midwifery personnel (24.40 per 10 000 population).^10^
*SCALA participating municipalities*	Intervention: Soacha (population: 93.154; located in metropolitan area of Bogota, part of department of Cundinamarca).^1^	Intervention: Tllapan (650.567)*, Benito Juárez (385.439), Álvaro Obregón (727.034); all one of 16 municipalities of Mexico City.^2^	Intervention: Callao (pop: 451.260): Provincial capital and one of the seven districts in Callao province, part of Callao region. Located at the West area of Lima, and borders the Pacific ocean.^3^
	Control: Funza (pop: 112.254), Madrid (93.154); both located in Western Savanna Province and part of the department of Cundinamarca, 25 km outside Bogota.^1^	Control: Miguel Hidalgo (372.889), Xochimilco (415.007), both one of 16 municipalities of Mexico City.^2^	Control: Chorillos (314.241) and Santiago de Surco (329.152); both one of the 43 districts of Lima province, located in Lima region, bordering eachother.^3^
		*two of PHCUs from this municipality are in control arm	

1 DANE (2018). Censo nacional de población y vivienda. Proyecciones de población. Available from: https://www.dane.gov.co/index.php/estadisticas-por-tema/demografia-y-poblacion/proyecciones-de-poblacion [accessed 23.9.2020]

2 INEGI (n.d.). Banco de indicadores, 2015. Available from https://www.inegi.org.mx/app/indicadores/?t=0070&ag=09014##D00700060 [accessed 23.9.2020]

3 INEI (2017). Censos nacionales 2017: XII Censo de Población, VII de Vivienda y III de Comunidades Indígenas. Sistema de Consulta de Base de Datos. Available from: http://censos2017.inei.gob.pe/redatam/ [accessed 23.9.2020]

4 OECD (2015). OECD Reviews of Health Systems: Colombia 2016. Paris: OECD Publishing.

5 Báscolo, E., Houghton, N., & Del Riego, A. (2018). Lógicas de transformación de los sistemas de salud en América Latina y resultados en acceso y cobertura de salud. *Revista Panamericana de Salud Pública*, *42*, e126.

6 WHO (n.d.) Global Health Expenditure database: https://apps.who.int/nha/database/ [accessed 7.10.2020]

7 WHO (2017). Primary health care systems (PRIMASYS): case study from Mexico, abridged version. Geneva: World Health Organization.

8 WHO (2017). Primary health care systems (PRIMASYS): case study from Peru, abridged version. Geneva: World Health Organization.

9 WHO (2017). Primary health care systems (PRIMASYS): case study from Colombia, abridged version. Geneva: World Health Organization.

10 WHO (n.d.) Global Health Workforce Statistics, the 2018 update, Available from: https://apps.who.int/gho/data/node.main.HWFGRP?lang=en [accessed 7.10.2020]

### Participants

In order to ensure the information was gathered from participants who were familiar with the intervention and/or setting, only stakeholders from the three countries who fulfilled at least one of the following inclusion criteria were invited to participate in the study: experience in the field of alcohol (prevention), experience in implementing any kind of intervention in PHC, currently working in a PHC centre. In each country, a local research group with knowledge of the local context identified the stakeholders in their network fitting these criteria and invited them to take part in the survey via e-mail. Both health professionals and professionals from other occupations (eg, regional health administrators) were invited to participate in the survey. Eighty-three stakeholders were invited to participate and in total; 55 stakeholders responded to the survey (66% response rate): 16 from Colombia (53% response rate), 18 from Mexico (75% response rate) and 21 (72% response rate) from Peru.

### Instrument

The survey was disseminated online and questions covered demographic characteristics (gender, country, occupation) and 24 items regarding appropriateness and barriers of alcohol SBA. All the survey questions were developed by the authors, as no instruments based on the TICD framework to study implementation outcomes and barriers were found in the literature.

*Appropriateness* was assessed with three questions covering: fit of intervention to the problem, fit to the local setting and fit of the provider. Respondents were asked to rate their agreement with alcohol SBA being an appropriate approach to reduce heavy alcohol use, and the PHC centre being a suitable setting to conduct alcohol SBA on 5-point Likert scales (1 = completely disagree to 5 = completely agree). Additionally, they had to indicate which health professionals they considered suitable to carry out alcohol SBA in primary care.

The development of a list of *barriers* to the implementation of SBA was guided by the TICD framework (Flottorp *et al.*, 2013), based on prior research identified through an examination of reviews in this area (Johnson *et al.*, 2011; O’Donnell, *et al.*, 2014; Derges *et al.*, 2017), and on recommendations of an expert panel with experience in the topic. The barriers identified in the literature have been extracted and categorised in the TICD framework under relevant domains and determinant headings. The list was shared with the expert panel, which selected additional determinants considered important based on their knowledge and experience. The full list of barrier items based on literature review and expert panel recommendations consisted of 46 items. This initial list was then shared with the local research teams in the three countries. Based on their feedback, the full list was shortened in order to increase the likelihood of response. Next, the most relevant determinants were selected by the central research team based on consultation with the local research teams in the three countries. The final, shortened list contained 21 items, with each categorised into the corresponding TICD framework determinants in one of the domains: guideline factors, individual health professional factors, patients factors, professional interactions, incentives and resources, capacity for organisational change, social legal and political factors. Questions were rated on a 5-point Likert scale (1 = completely disagree, it is not a barrier to 5 = completely agree, it is a large barrier). Both the long and shortened lists of barriers are available as supplementary material.

The survey was developed in English, translated to Spanish, and further refined based on feedback from the local research teams. Before dissemination, two to three experts per country piloted the survey.

### Data collection

The data were collected in April and May 2019 using Formdesk, an online survey software. Respondents were invited to participate through e-mail by the local researcher and were sent a reminder after a week in case of no response. No identifiable data were collected, and the survey was anonymous. Participants had to sign the informed consent electronically before they were able to participate in the survey. Ethical review was not required for anonymous online surveys in all three countries.

### Data analysis

IBM SPSS Statistics 24 was used for data analysis. Data was first analysed separately for each of the countries (Colombia, Mexico, Peru), and for barriers, also by occupation. To obtain the percentages of respondents agreeing with the statements, the number of participants agreeing or completely agreeing with an item were divided by the number of all participants. Medians and interquartile ranges were computed. Due to the small sample size and non-normal distribution, as tested with one-way Kolmogorov–Smirnov test, non-parametric tests (Kruskal–Wallis *H* for medians and Chi square for percentages) were used for comparisons. Where additional post-hoc tests (Mann–Whitney *U*) were used, Bonferroni correction was applied.

## Results

In total, 55 respondents participated in the survey. Their demographic characteristics are presented in Table 2. Approximately half of the participants were health care providers, out of which the majority were general practitioners (GPs) and psychologists.

**Table 2.** Characteristics of key local stakeholders included in the study

	Overall		Colombia		México		Perú	
	*n*	%	*n*	%	*n*	%	*n*	%
*Country*								
Colombia	16	29.09						
México	18	32.73						
Perú	21	38.18						
*Gender*								
Female	34	61.82	13	81.25	8	44.44	13	61.90
Male	21	38.18	3	18.75	10	55.56	8	38.10
*Occupation*								
Health care provider	28	50.91	9	56.25	6	33.33	13	61.90
GP	12	21.82	4	25.00	2	11.11	6	28.57
Psychologist	14	25.45	5	31.25	4	22.22	5	23.81
Other health care provider*	2	3.64	0	0.00	0	0.00	2	9.52
Other occupations	26	47.27	7	43.75	12	66.67	7	33.33
Civil servant	8	14.55	3	18.75	4	22.22	1	4.76
Civil society representative	8	14.55	1	6.25	3	16.67	4	19.05
Academic/researcher	6	10.91	2	12.50	4	22.22	0	0.00
Other**	4	7.26	1	6.25	1	5.56	2	9.52
Unknown	1	1.82	0	0.00	0	0.00	1	4.76

* midwife, social worker.

** PHC centre manager, national public policy advisor, national consultant and private treatment centre employee.

### Appropriateness

As seen in Table 3, there were high proportions of respondents (75% or above, with one exception) considering alcohol SBA to be an appropriate approach to reduce heavy alcohol use (fit to the problem), and the PHC centre being a suitable place to perform alcohol SBA (fit to the setting). Considering the fit of provider, respondents in all three countries indicated four types of professionals to be appropriate to carry out alcohol SBA (all percentages above 80%): GPs, nurses, psychologists and social workers.

**Table 3.** Response rates and comparison of perceived appropriateness of alcohol SBA in Colombia, México and Perú

	% Agree*			Comparison			
	Colombia	México	Perú	Colombia	México	Perú	
	*n* = 16	*n* = 18	*n* = 21	Me (IQR)^†^	Me (IQR)	Me (IQR)	*P* **
Consider alcohol SBA is an appropriate approach to reduce heavy alcohol use	87.50	77.78	57.14	5.00 (1.00)	4.50 (1.25)	4.00 (1.50)	0.01^a^
Consider PHC centre is a suitable place to carry out alcohol SBA	100.00	83.33	76.19	5.00 (0.75)	5.00 (1.00)	4.00 (1.50)	0.10
Providers considered suitable to carry out alcohol SBA in primary health care:							
GP	93.75	94.44	80.95				0.31
Nurse	87.50	77.78	90.48				0.51
Psychologist	93.75	100.00	95.24				0.59
Social worker	87.50	94.44	85.71				0.66
Midwife	37.50	38.89	52.38				0.59
Other	12.50	33.33	14.29				0.22

† Me–Median, IQR-Interquartile range.

* % summed responses Agree and Completely agree for the first two rows, % Yes for the latter six rows.

** Kruskal–Wallis *H* test for the first two rows, Chi square test for the latter six rows.

a Post-hoc test showed significant difference between Peru and Colombia (Mann–Whitney *U* = 15.440, *P* = 0.007).

Kruskal–Wallis *H* test showed a significant difference between countries’ perception of alcohol SBA as an appropriate approach to reduce heavy alcohol use, with post-hoc tests revealing a significant difference between Colombian (most endorsements) and Peruvian respondents (least endorsements). No other county differences were found.

### Barriers to implementation of alcohol SBA

In Table 4, the percentages concerning perceived barriers for implementation are presented for all the three countries, as well as medians and their comparisons. Four barriers stood out with having high rating (defined as two thirds or more of respondents) in all three countries: heavy drinking patients’ beliefs that their drinking is normal (patient factors TICD domain), lack of on-going support for providers (assistance for clinicians TICD domain), difficulty of accessing referral services (professional interactions TICD domain) and lenient laws and regulations influencing price and availability that encourage cultural tolerance to alcohol (social, political and legal factors TICD domain).

**Table 4.** Response rates and comparison of perceived barriers to alcohol SBA by country

	TICD Determinant of practice		% Agree*			Comparison			
			Colombia	México	Perú	Colombia	México	Perú	
TICD Domain^×^			*n* = 16	*n* = 18	*n* = 21	Me (IQR)^†^	Me (IQR)	Me (IQR)	*P* **
1. Guideline factors	Clarity	Guidelines for screening and giving advice for heavy drinking are not clear enough	31.25	5.56	42.86	2.00 (3.00)	2.00 (2.00)	3.00 (1.00)	0.001^a^
	Effort	Screening and giving advice for heavy drinking is too much work to do	12.50	11.11	19.05	2.00 (1.00)	1.00 (1.00)	2.00 (2.00)	0.50
	Feasibility	Screening and giving advice for heavy drinking in our everyday practice is not feasible	6.25	16.67	14.29	2.00 (1.00)	2.00 (2.00)	2.00 (1.00)	0.89
	Cultural appropriateness	Screening and giving advice for heavy drinking is not appropriate in our culture	6.25	11.11	4.76	1.00 (1.00)	1.00 (1.00)	2.00 (1.00)	0.16
2. Individual health professional factors	Skills needed to adhere	Providers do not have the skills to implement screening and brief advice programmes for heavy drinking	62.50	50.00	47.62	4.00 (2.75)	3.50 (3.00)	3.00 (2.50)	0.84
	Expected outcome	Providers think that screening and giving advice for heavy drinking will not help their patients	56.25	44.44	42.86	4.00 (2.00)	3.00 (1.00)	3.00 (2.00)	0.84
	Intention and motivation	Providers consider that screening and giving advice for heavy drinking is not their responsibility	50.00	61.11	57.14	3.50 (2.00)	4.00 (1.5)	4.00 (2.00)	0.91
	Self-efficacy	Providers believe they cannot help their heavy drinking patients	56.25	44.44	61.90	4.00 (1.75)	3.00 (2.00)	4.00 (2.00)	0.93
	Emotions	Providers are reluctant to screen for heavy drinking due to social and cultural barriers	56.25	50.00	61.90	4.00 (2.00)	3.50 (2.25)	4.00 (1.00)	0.83
	Capacity to plan change	Providers do not have enough time to screen and give advice for heavy drinking	87.50	61.11	47.62	4.00 (1.00)	4.00 (3.00)	3.00 (2.00)	0.08
3. Patient factors	Patient beliefs and knowledge	Most heavy drinking patients think that their drinking is normal	93.75	72.22	80.95	4.00 (0.75)	4.00 (2.00)	4.00 (1.00)	0.89
	Patient preferences	Patients do not like to discuss their alcohol consumption with their doctor or nurse	43.75	61.11	71.43	3.00 (2.00)	4.00 (2.00)	4.00 (1.50)	0.41
4. Professional interactions	Referral processes	There are difficulties with access to referral services for patients with alcohol problems	81.25	77.78	76.19	4.00 (1.00)	4.00 (1.25)	4.00 (2.00)	0.74
5. Incentives and resources	Availability of necessary resources	Instruments for screening and giving advice to heavy drinkers do not exist	12.50	11.11	38.10	1.50 (1.00)	1.00 (1.25)	3.00 (2.00)	0.008^b^
	Financial incentives and disincentives	There is lack of financial incentives for providers to carry out screening and advice	68.75	66.67	42.86	4.00 (2.00)	4.00 (1.50)	3.00 (2.00)	0.32
	Nonfinancial incentives and disincentives	There is lack of non-financial incentives for providers to carry out screening and advice	75.00	61.11	66.67	4.00 (0.75)	4.00 (1.00)	4.00 (1.00)	0.84
	Assistance for clinicians	There is lack of on-going support for providers to carry out screening and advice	93.75	77.78	95.24	4.00 (0.00)	4.00 (0.25)	4.00 (1.00)	0.17
6. Capacity for organisational change	Capable leadership	There is lack of support by the leadership in PHC centres to support and implement programmes of screening and advice	43.75	55.56	57.14	3.00 (1.75)	4.00 (1.00)	4.00 (1.50)	0.36
	Assistance for organisational changes	There is lack of necessary organizational changes in PHC centres to implement screening and advice	56.25	66.67	80.95	4.00 (1.75)	4.00 (1.00)	4.00 (1.00)	0.11
7. Social, political and legal factors	Economic constraints on the health care budget	There is lack of sufficient staff in PHC centres to be able to implement programmes for screening and advice	50.00	44.44	76.19	3.5 (2.00)	3.00 (2.00)	4.00 (1.00)	0.08
	Legislation	Laws and regulations in the country that influence the price and availability of alcohol are too lenient, encouraging cultural tolerance to alcohol	93.75	66.67	90.48	4.00 (1.00)	4.00 (2.25)	4.00 (1.00)	0.63

× Domains 3–7 can also be considered as contextual factors, based on (Nilsen and Bernhardsson, 2019).

† Me–Median, IQR-Interquartile range.

* % responses Agree and Completely agree.

** Kruskal–Wallis *H* test.

a Post-hoc test showed significant difference between Mexico and Peru (Mann–Whitney *U* = −18.10, *P* = 0.001).

b Post-hoc test showed significant difference between Mexico and Peru (Mann–Whitney *U* = −13.56, *P* = 0.018) and Colombia and Peru (Mann–Whitney *U* = −12.82, *P* = 0.035).

Three barriers had high ratings in two countries: lack of financial (Colombia and Mexico) and non-financial incentives (Colombia and Peru) (both Incentives and Resources TICD domain), and lack of necessary organisational changes (Mexico and Peru) (Capacity of organisational change TICD domain). Certain barriers with high agreement percentages were also country specific: lack of sufficient staff for implementation in the centre as well as patients’ preference not to discuss their alcohol consumption in Peru (the first, social, political and legal factors and the latter, patient factors TICD domain) and lack of providers’ time in Colombia (individual health professional factors TICD domain). The barriers of SBA not being culturally appropriate, not feasible in practice and requiring too much effort (all in Guideline factors TICD domain) were lowest rated in all three countries, with most percentages under 20%.

Country comparison showed two barriers with a statistically significant difference in their ratings: the guidelines for screening and brief advice not being clear enough and instruments for screening not being available. Post-hoc tests indicated that Peruvian respondents were more likely to endorse lack of guideline clarity as compared to Mexican respondents, and more likely to cite lacking availability of SBA instruments as a barrier compared to both Colombian and Mexican respondents. Despite the differences, those were not the most frequently endorsed barriers.

As health professional level barriers are commonly mentioned in previous qualitative research in this area for example (Johnson *et al.*, 2011; Derges *et al.*, 2017), but were not among the highest rated barriers in our survey (with agreement percentages between 42 and 62%), we decided to further explore barriers by occupation. The available sample allowed us to compare GPs’ responses with responses from psychologists and other occupations (non-health care providers). Comparison showed statistically significant differences in three determinants from the individual health professional factors TICD domain: lack of skills to implement the intervention, providers thinking that alcohol SBA will not help their patients and not considering providing alcohol SBA as their responsibility (Table 5). In all three cases, the GPs rated these barriers significantly lower than psychologists and other professionals.

**Table 5.** Response rates and comparison of perceived barriers to alcohol SBA by occupation

	TICD Determinant of practice		% Agree*			Comparison			
			GP	Psycholo-gist	Other occupation	GP	Psycholo-gist	Other occupation	
TICD Domain^×^			*n* = 12	*n* = 14	*n* = 26	Me (IQR)^†^	Me (IQR)	Me (IQR)	*P* **
1. Guideline factors	Clarity	Guidelines for screening and giving advice for heavy drinking are not clear enough	41.67	21.43	23.08	2.50 (2.75)	3.00 (2.25)	2.00 (2.25)	0.56
	Effort	Screening and giving advice for heavy drinking is too much work to do	25.00	7.14	15.38	2.00 (2.75)	1.00 (1.00)	2.00 (2.00)	0.28
	Feasibility	Screening and giving advice for heavy drinking in our everyday practice is not feasible	16.67	7.14	15.38	2.00 (1.75)	1.50 (1.00)	2.00 (1.25)	0.48
	Cultural appropriateness	Screening and giving advice for heavy drinking is not appropriate in our culture	0.00	7.14	11.54	1.50 (1.00)	1.00 (1.00)	1.50 (1.00)	0.75
2. Individual health professional factors	Skills needed to adhere	Providers do not have the skills to implement screening and brief advice programmes for heavy drinking	25.00	78.57	53.85	1.50 (2.5)	4.00 (3.00)	4.00 (2.00)	0.03^a^
	Expected outcome	Providers think that screening and giving advice for heavy drinking will not help their patients	8.33	57.14	61.54	2.00 (0.00)	4.00 (3.00)	4.00 (1.00)	0.00^b^
	Intention and motivation	Providers consider that screening and giving advice for heavy drinking is not their responsibility	0.00	57.14	80.77	1.00 (1.00)	4.00 (3.00)	4.00 (0.00)	0.00^c^
	Self-efficacy	Providers believe they cannot help their heavy drinking patients	25.00	57.14	65.38	2.00 (1.75)	4.00 (3.00)	4.00 (1.25)	0.1
	Emotions	Providers are reluctant to screen for heavy drinking due to social and cultural barriers	33.33	57.14	65.38	2.50 (2.00)	4.00 (4.00)	4.00 (2.00)	0.16
	Capacity to plan change	Providers do not have enough time to screen and give advice for heavy drinking	50.00	85.71	61.54	3.00 (2.75)	4.00 (4.00)	4.00 (1.25)	0.16
3. Patient factors	Patient beliefs and knowledge	Most heavy drinking patients think that their drinking is normal	58.33	85.71	88.46	4.00 (2.75)	4.00 (4.00)	4.00 (1.00)	0.29
	Patient preferences	Patients do not like to discuss their alcohol consumption with their doctor or nurse	33.33	78.57	57.69	3.00 (2.00)	4.00 (4.00)	4.00 (2.00)	0.14
4. Professional interactions	Referral processes	There are difficulties with access to referral services for patients with alcohol problems	66.67	78.57	88.46	4.00 (3.00)	4.00 (4.00)	4.00 (1.00)	0.84
5. Incentives and resources	Availability of necessary resources	Instruments for screening and giving advice to heavy drinkers do not exist	33.33	14.29	19.23	1.50 (3.00)	2.00 (2.00)	2.00 (2.00)	0.97
	Financial incentives and disincentives	There is lack of financial incentives for providers to carry out screening and advice	58.33	64.29	61.54	4.00 (2.75)	4.00 (3.25)	4.00 (2.00)	0.84
	Nonfinancial incentives and disincentives	There is lack of non-financial incentives for providers to carry out screening and advice	66.67	78.57	65.38	4.00 (2.75)	4.00 (4.00)	4.00 (1.00)	0.28
	Assistance for clinicians	There is lack of on-going support for providers to carry out screening and advice	83.33	92.86	88.46	4.00 (1.00)	4.00 (3.00)	4.00 (0.00)	0.82
6. Capacity for organisational change	Capable leadership	There is lack of support by the leadership in PHC centres to support and implement programmes of screening and advice	66.67	28.57	61.54	4.00 (2.50)	3.00 (3.00)	4.00 (1.00)	0.22
	Assistance for organisational changes	There is lack of necessary organizational changes in PHC centres to implement screening and advice	66.67	78.57	65.38	4.00 (2.50)	4.00 (3.00)	4.00 (1.00)	0.98
7. Social, political and legal factors	Economic constraints on the health care budget	There is lack of sufficient staff in PHC centres to be able to implement programmes for screening and advice	41.67	64.29	61.54	3.00 (3.00)	4.00 (3.00)	4.00 (2.00)	0.91
	Legislation	Laws and regulations in the country that influence the price and availability of alcohol are too lenient, encouraging cultural tolerance to alcohol	83.33	92.86	80.77	4.00 (1.00)	5.00 (4.00)	4.00 (1.00)	0.07

× Domains 3–7 can also be considered as contextual factors, based on (Nilsen and Bernhardsson, 2019).

† Me–Median, IQR-Interquartile range.

* % responses Agree and Completely agree.

** Kruskal–Wallis *H* test.

a Post-hoc test showed significant difference between GPs and psychologists (Mann–Whitney *U* = −14.69, *P* = 0.023).

b Post-hoc test showed significant difference between GPs and psychologists (Mann–Whitney *U* = −16.62, *P* = 0.009) and GPs and other occupations (Mann–Whitney *U* = −19.72, *P* = 0.001).

c Post-hoc test showed significant difference between GPs and psychologists (Mann–Whitney *U* = −19.05, *P* = 0.002) and GPs and other occupations (Mann–Whitney *U* = −22.91, *P* = 0.001)

## Discussion

The aim of this study was to assess and compare the perceived general appropriateness of alcohol screening and brief advice and the perceived barriers to its implementation from the perspective of local stakeholders in three municipalities in Colombia, Mexico and Peru.

The study showed that delivering alcohol SBA in PHC setting was generally seen as an appropriate intervention to reduce heavy alcohol use in these three Latin American countries, although there were small differences, with SBA being considered more appropriate among Colombian compared to Peruvian respondents. In all three countries, GPs, nurses, psychologists and social workers were considered suitable for delivery of SBA in primary care. This suggests that scaling up SBA programmes in PHC in the Latin American context might be achieved by expanding the range of providers. Whilst many studies from HIC have shown the effectiveness of SBA with GPs as the intervention provider (O’Donnell *et al.*, 2014), there is also emerging evidence of effectiveness of non-physician led alcohol interventions (Sullivan *et al.*, 2011), such as nurses (Platt *et al.*, 2016) or social workers in social service settings (Schmidt *et al.*, 2015). Another consideration not explored in the study, but relevant for practice and further investigation, is the possibility of interprofessional approaches, where team members of different occupations work together to improve health outcomes for the patient (Zwarenstein *et al.*, 2005). In case of alcohol screening in brief advice this could mean screening done by one member of the team (eg, nurse) and advising by another (eg, GP or psychologist). This could enable scaling up via better integration of SBA into the existing workflow. Further research is needed however on the effectiveness and patient acceptability of SBA delivered by non-physicians in the LMIC context.

The assessment of barriers also showed that the pattern in perception of barriers was similar in all three countries. This implies that a similar approach can be used to implement alcohol SBA across these particular countries, with tailoring efforts focussed on the specific parts needed to improve fit in the local context. In general, intervention-related factors (guideline factors TICD domain) such as lack of feasibility or cultural fit were not seen as major barriers, which echo previous evidence from the HIC context. Yet countries differed concerning SBA guideline clarity: at least a third of Colombian and Peruvian respondents mentioned lack of clarity as a barrier; whereas the percentage among Mexican respondents was much lower. This reflects the differing national contexts with regard to the existing guidelines: in Mexico, official standards establish the obligatory procedures and criteria for mandatory prevention, treatment and control of addictions, which include asking questions on alcohol use (*Norma Oficial Mexicana NOM-028-SSA2-2009 para la prevención, tratamiento y control de las adicciones*, 2009), and including this information in the patient’s history (*Norma Oficial Mexicana NOM-004-SSA3-2012 del expediente clínico*, 2012), specifically in primary health care context. In Colombia, the alcohol SBA recommendations are included as part of clinical practice guidelines that focus on detection and treatment of alcohol abuse and dependence on primary, secondary and tertiary care level (Ministerio de Salud y Protección Social, 2013), but there are no official standards as in Mexico. Finally, in Peru, recommendation for providers to deliver alcohol screening can be considered implicitly included in general recommendations to perform mental health-related screening (alcohol use disorder being considered as one of subcategories) (Ministerio de Salud Peru, 2018), therefore making the alcohol SBA guidelines potentially less clear. However, when considered in light of other higher rated barriers, improving clarity of guidelines (at least in Colombia and Peru) is not the main priority.

Looking at the results from the perspective of the TICD framework, the barriers with the highest agreement in all countries can be categorised as contextual (as defined in Nilsen and Bernhardsson, 2019). Specifically, respondents in all three countries highlighted heavy drinking patients’ thinking that their drinking is normal, lack of on-going support for providers, difficulty of accessing referral services and lenient laws and regulations influencing price and availability encouraging cultural tolerance to alcohol, as key factors affecting implementation. Again, these barriers reflect those identified in HIC literature, where patients’ normalisation of heavy drinking, referral issues and organisational factors, including lack of a supportive policy environment, are commonly cited as obstacles to delivery (Anderson *et al.*, 2003; Johnson *et al.*, 2011; Derges *et al.*, 2017; Vendetti *et al.*, 2017). To tackle the barrier of patients’ normalised perception of their own heavy drinking, there is a need for communication strategies surrounding SBA programmes to involve a reframing component, which highlights that much alcohol-related harm is experienced by those drinking at non-dependent levels (eg, see (Heather, 2006). Lack of restrictions for on/off premise sales of alcoholic beverages or limited restrictions on alcohol advertising in the participating countries might have contributed to the perception of lenient alcohol control policies expressed by the stakeholders in this survey (World Health Organisation, 2018). Indeed, recent research has highlighted the need to address these types of policy factors in LMICs in order to reduce alcohol-related harm (Shield *et al.*, 2020).

Barriers from the individual health professional factors TICD domain were neither among the highest nor among the lowest rated barriers. This might have been influenced by differing opinions based on occupation, as shown by the comparison between GPs, psychologists and others. The provider related factors such as lack of skills, lack of responsibility and belief about the intervention not helping the patients were considered much less of a barrier by the GP respondents compared to psychologists and other occupations. Studies from HIC countries however suggest that attitudinal factors do hinder GPs’ implementation of SBA, such as lower role security and therapeutic commitment (Anderson *et al.*, 2003), as well as aligning with the disease rather than preventive model of work and valuing individual personal responsibility for protection from alcohol-related harm (Anderson *et al.*, 2014). Whilst the sample is too small to draw definite conclusions, some of the possible reasons for our results may be selection bias (ie, GPs participating in the survey were potentially already more educated and aware about alcohol), GP’s higher self-efficacy when it comes to delivering interventions in PHC, or psychologists seeing the brevity of the intervention as less appropriate to their practice. Nevertheless, these preliminary results point us in direction of the health professional-related barriers potentially being profession-specific and suggest that more research is needed to explore the perspectives of and barriers experienced by other occupations.

Results of this study suggest that multi-level strategies are needed to address barriers to widespread SBA implementation in Colombia, Mexico and Peru. First, although individual health professional level factors were not ranked highest, barriers relating to a perceived lack of skills, self-efficacy, role-legitimacy or and belief in intervention effectiveness can be addressed through means of provider training programmes. The preliminary differences found here between GPs and psychologists suggest that tailoring training might be necessary, using different approaches for providers of different occupations, based on the specific needs, as well as specific strengths, of different health care providers (Wamsley *et al.*, 2018).

Yet, whilst training can help increase providers’ intervention-related knowledge, skills and self-efficacy, previous research has shown that is unlikely to be sufficient to improve implementation on its own, particularly over the longer term (Anderson, 2004). Looking at the TICD domains of the highest rated barriers in this study, it can be seen that they all relate to the wider social, political and cultural SBA delivery context. Thus, interventions that provide continuous support for the providers (Anderson *et al.*, 2016) and efforts to change the community social norms (Anderson *et al.*, 2018) related to alcohol (through education or legislation) are also needed to address the perceived relevant barriers in these three countries. This has been shown also through previous work in HIC, where series of multi-country studies concluded that education and support in the working environment are necessary to increase involvement of health care providers (in that case GPs) in managing alcohol problems (Anderson *et al.*, 2003, 2014).

### Strengths and weaknesses

This study contributes to the literature on SBA implementation with evidence from an underexplored region (Latin America) using a quantitative approach that allows for direct comparisons between three countries. The list of barriers to implementation of alcohol SBA was developed within a theoretical framework, combining evidence from previous empirical studies and recommendations from an expert panel. Furthermore, inclusion of a range of key local stakeholders with different occupations and experience in the topic allowed for a broader perspective on barriers to implementation, assessing determinants on various professional and health system levels. We encourage the use of the proposed list of barriers in future SBA barrier assessments in PHC or other occupations across Latin America and elsewhere, if locally adapted.

Beside the abovementioned strengths, the current study also has limitations. One, due to its focus on a municipal context in three Latin American countries and a limited range of eligible stakeholders with enough experience to be consulted, the low sample size limits broader generalisation of the results. Additionally, as the study focussed only on the three countries participating in SCALA project, the results cannot necessarily be generalised to other Latin American countries. While comparison between the three countries points to predominant similarities rather than differences in barriers perception, further local assessment would be necessary before scaling up alcohol SBA beyond Colombia, Mexico and Peru. Two, there are also some general shortcomings of the survey approach to identifying barriers that should be acknowledged: whilst this approach enables us to compare statistically the relative importance of specific barriers to implementation, as these barriers were pre-determined by the team constructing the questionnaire, some other relevant barriers might have been overlooked (Nilsen, 2015). In our case, the list of barriers had to be considerably shortened in its final form in order to ensure respondents’ completion of the survey, resulting in potentially relevant barrier(s) being excluded. However, it is important to note that this shortcoming was addressed by consulting with the experts and local research partners when determining the final list. Three, the perceived barriers may not necessarily correspond to the actual barriers encountered when implementing the intervention (Nilsen, 2015). This was beyond the scope of our study, but our findings provide a useful baseline data, whereby future intervention evaluations can compare the encountered barriers to the perceived ones identified in our study. Four, this study did not look at the patient perspective on the implementation of alcohol SBA, which should also be explored in further studies, in line with previous research, such as Lock, 2004, or Hutchings *et al.*, 2006. Furthermore, among health professionals our sample predominantly contained perspectives of GPs and psychologists and further perspective from other professionals also considered appropriate to deliver alcohol SBA (nurses and social workers) should be included in any follow-up research.

### Future perspectives

Findings of the study point to the necessity of considering barriers on a broader scale than just at the individual provider level. For SCALA project, this means designing process evaluation related data collection in a way to capture the broad spectrum of possible experienced barriers and facilitators. Results will also be used along other data collected in the SCALA project to help explain the outcome on provider level – why did or did not providers implement alcohol SBA in their daily practice. Results may also contribute to wider implementation of alcohol SBA in Latin American countries. We encourage other researchers and practitioners to use the developed instrument (available as the supplementary material) for rapid assessment of appropriateness and barriers in any novel LMIC context and as an aid when tailoring the intervention to the specific local context.

## Conclusion

This study investigated local stakeholders’ views of the appropriateness of alcohol SBA, as well as their perceived barriers to its implementation in three municipalities in Colombia, Mexico and Peru. Implementation of SBA in PHC is generally considered as an appropriate means to reduce alcohol-related harm in all three countries. In contrast to evidence from HIC countries, context-related factors were cited as major barriers to SBA implementation, namely lack of support for providers, difficulties with accessing referral services, patients underestimating the danger of their consumption levels and lax alcohol control legislation. Despite the similarities, it is still necessary to be sensitive to existing differences and tailor of the specific SBA programmes for each country.

## Supplementary material

For supplementary material accompanying this paper visit https://doi.org/10.1017/S1463423620000675. click here to view supplementary material
