# Supplementary material for: Perceived appropriateness of alcohol screening and brief advice programmes in Colombia, Mexico and Peru and barriers to their implementation in primary health care – a cross-sectional survey
Source: Prim Health Care Res Dev. 2021 Jan 28;22:e4. doi: 10.1017/S1463423620000675 (PMC8057507; doi:10.1017/S1463423620000675)
Supplement: Supplementary file 1 [file S1463423620000675sup001.docx]

**I. Initial list of barriers categorized in the TICD framework**

Table 1: Overview of TICD framework determinants and the initially selected items

| **TICD framework - domains** | **TICD framework - subdomains** | **TICD framework determinants** | **Selected based on literature review** | **Selected by the expert panel** | **Item description** | **Final chosen item** |
| --- | --- | --- | --- | --- | --- | --- |
| Guideline factors | Recommendation | Quality of evidence supporting recommendation |  |  |  |  |
|  |  | Strength of recommendation |  |  |  |  |
|  |  | Clarity | x |  | Guidelines for screening and giving advice for heavy drinking are not clear enough | x |
|  |  |  | x |  | The guidelines on how to perform alcohol screening and brief interventions that exist are poorly designed |  |
|  |  | Cultural appropriateness |  | x | Screening and giving advice for heavy drinking is not appropriate in our culture | x |
|  |  | Accessibility of recommendation |  |  |  |  |
|  |  | Source of the recommendation |  |  |  |  |
|  |  | Consistency with other guidelines |  |  |  |  |
|  | Recommended clinical intervention | Feasibility |  | x | Screening and giving advice for heavy drinking in our everyday practice is not feasible | x |
|  |  | Accessibility of the intervention | x |  | There is lack of suitable materials that could be used for alcohol screening and brief interventions |  |
|  | Recommended behaviour | Compatibility | x |  | The approach of screening and brief intervention to address heavy alcohol use is not compatible with current primary care practice |  |
|  |  | Effort | x |  | Screening and giving advice for heavy drinking is too much work to do | x |
|  |  | Trialability | x |  | There is lack of appropriate training for providers on how to do alcohol screening and brief interventions |  |
|  |  | Observability |  |  |  |  |
| Individual health professional factors | Knowledge and skills | Domain knowledge | x |  | Providers do not know enough about risky drinking and harmful alcohol use / impact of alcohol on health |  |
|  |  | Awareness and familiarity with the recommendation | x |  | Providers are not familiar with the screening tools and content of the brief intervention |  |
|  |  | Knowledge about own practice |  |  |  |  |
|  |  | Skills needed to adhere |  | x | Providers do not have the skills to implement screening and brief advice programs for heavy drinking | x |
|  | Cognitions (including attitudes) | Agreement with the recommendation | x |  | Providers view alcohol as delicate subject to raise in consultation |  |
|  |  |  | x |  | Alcohol prevention is not priority for providers |  |
|  |  | Attitudes towards guidelines in general |  |  |  |  |
|  |  | Expected outcome | x |  | Providers think doing alcohol screening and brief intervention will have negative impact on their relationship with the patient |  |
|  |  |  | x |  | Providers think that screening and giving advice for heavy drinking will not help their patients | x |
|  |  |  | x |  | Providers are concerned they might offend patients by discussing drinking |  |
|  |  | Intention and motivation | x |  | Providers are reluctant to discuss drinking unless signs of risk are apparent |  |
|  |  |  | x |  | Providers consider that screening and giving advice for heavy drinking is not their responsibility | x |
|  |  | Self-efficacy | x |  | Providers believe they cannot help their heavy drinking patients | x |
|  |  | Learning style |  |  |  |  |
|  |  | Emotions | x |  | Providers believe that patients are not interested in getting help regarding their alcohol use. |  |
|  |  |  | x |  | Providers feel that because of their own drinking habits they are not entitled to give advice |  |
|  |  |  | x |  | Providers are anxious they will give wrong advice |  |
|  |  |  | x |  | Providers do not think it is their role to address patients’ alcohol use |  |
|  |  |  | x |  | Providers are afraid they would stigmatize a patient by asking about his alcohol use |  |
|  |  |  | x |  | Providers are reluctant to screen for heavy drinking due to social and cultural barriers | x |
|  | Professional behaviour | Nature of behaviour |  |  |  |  |
|  |  | Capacity to plan change | x |  | Providers do not have enough time to screen and give advice for heavy drinking | x |
|  |  |  | x |  | Providers find it difficult to implement alcohol screening and brief intervention in normal flow of work |  |
|  |  | Self-monitoring or feedback |  |  |  |  |
| Patient factors |  | Patient needs |  |  |  |  |
|  |  | Patient beliefs and knowledge | x |  | Most heavy drinking patients think that their drinking is normal | x |
|  |  | Patient preferences |  | x | Patients do not like to discuss their alcohol consumption | x |
|  |  | Patient motivation |  |  |  |  |
|  |  | Patient behaviour | x |  | Patients would react in a negative way to the intervention |  |
| Professional interactions |  | Communication and influence |  |  | There is poor communication among the providers around alcohol related care |  |
|  |  | Team processes |  | x | There is lack of support for doing alcohol screening and brief interventions from other members of the provider’s team |  |
|  |  | Referral processes | x |  | There is nowhere to refer patients with alcohol problems | x |
| Incentives and resources |  | Availability of necessary resources | x |  | Instruments for screening and giving advice to heavy drinkers do not exist | x |
|  |  |  | x |  | There is limited availability of treatment resources |  |
|  |  | Financial incentives and disincentives |  | x | There is lack of financial incentives for providers to carry out screening and advice for heavy drinkers | x |
|  |  |  |  | x | There is lack of adequate financial support /incentives for the primary healthcare center managers to support the providers in carrying out alcohol screening and brief interventions |  |
|  |  | Nonfinancial incentives and disincentives |  | x | There is lack of non-financial incentives for providers to carry out screening and advice for heavy drinkers | x |
|  |  |  |  | x | There is lack of adequate non-financial incentives for the primary healthcare center managers to support the providers in carrying out alcohol screening and brief interventions |  |
|  |  | Information system |  |  |  |  |
|  |  | Quality assurance and patient safety systems |  |  |  |  |
|  |  | Continuing education system |  |  |  |  |
|  |  | Assistance for clinicians |  | x | There is lack of ongoing support for providers to carry out screening and advice for heavy drinkers | x |
| Capacity for organisational change |  | Mandate, authority and accountability for making necessary changes |  |  |  |  |
|  |  | Capable leadership |  | x | There is lack of leadership in primary health care centres to support and implement programmes of screening and advice for heavy drinkers | x |
|  |  | Relative strength of supporters and opponents |  | x | There are stakeholders (inside PHC) actively opposing implementation of alcohol screening and brief intervention in primary health care centers |  |
|  |  | Regulations, rules, policies |  | x | There exist organizational rules and policies that are hindering/in contradiction with alcohol screening and brief intervention implementation in the PHC |  |
|  |  | Priority of necessary change |  |  |  |  |
|  |  | Monitoring and feedback |  |  |  |  |
|  |  | Assistance for organisational changes | x |  | There is lack of support in primary health care centres to help make the necessary organizational changes to implement screening and advice for heavy drinking | x |
| Social, political and legal factors |  | Economic constraints on the health care budget |  | x | There is lack of sufficient staff in primary health care centres to be able to implement programmes for screening and advice for heavy drinkers | x |
|  |  | Contracts |  |  |  |  |
|  |  | Legislation |  | x | Laws and regulations in the country that influence the price and availability of alcohol are too lenient, *making the intervention less effective* | x |
|  |  | Payer or funder policies |  |  |  |  |
|  |  | Malpractice liability |  |  |  |  |
|  |  | Influential people | x |  | There are stakeholders (outside PHC) actively opposing implementation of alcohol screening and brief intervention in primary health care centers |  |
|  |  | Corruption |  |  |  |  |
|  |  | Political stability |  | x | There is lack of political stability on municipality / country level |  |

***II. Online questionnaire in Spanish***

Estimado encuestado,

Lo invitamos a participar en una investigación internacional sobre **la prevención del consumo excesivo de alcohol** in Sud America. Nos interesa saber su opinión sobre cuáles considera que son las barreras más importantes en su país **para la implementación de la detección del consumo de alcohol y consejo breve para reducir el consumo excesivo en la atención primaria.** Por esta razón, le agradecemos que dedique 10 minutos para completar este cuestionario.

Esta investigación es parte de proyecto **SCALA** ([https://www.scalaproject.eu/](https://www.scalaproject.eu/)), y cuenta con el apoyo financiero de la Union Europea y con **Instituto Nacional de Psiquiatria Ramon de la Fuente Muniz** in México, **Corporacion Nuevos Rumbos** in Colombia y **Universidad Peruana Cayetano Heredia** in Perú como principales socios de implementación in Sud America.

Sus respuestas nos ayudarán a enfrentar mejor las barreras en la práctica diaria in contexto de Sud America.

La encuesta es completamente anónima. Los resultados de la encuesta solo serán analizados de manera agregada. Los resultados agregados se discutirán y utilizarán para mejorar la implementación de esta intervención y se resumirán en una publicación/informe del proyecto.

- He comprendido completamente la información proporcionada y mi participación en la investigación es completamente voluntaria.

--------------------------------------------------------

Sexo

a) Mujer

b) Hombre

País

a) Colombia

b) México

c) Perú

Rol profesional

a) Profesional de la salud

b) Jefe de Centro de Atención Primaria en Salud (CAPS) o Centro de Primer Nivel de Atención

c) Trabajador administrativo (gubernamental, regional, municipal)

d) Representante de la sociedad civil

e) Académico

f) Otro: _________________________

Si usted es un profesional de la salud, ¿cuál es su profesión?

Médico/a

Enfermero/a ( jefe)

Psicólogo/a

Enfermero/a técnico/a

Trabajador/a social

Obstetra/ partera

Otro:

¿Tiene usted experiencia profesional previa en:

a) implementación de programas de prevención de problemas relacionados con el consumo de alcohol?

si / no

b) la implementación de programas preventivos (en general o sobre alcohol) en centros de atención primaria?

si / no

**1.General**

Las preguntas en esta investigación se refieren a la detección del consumo excesivo de alcohol en pacientes y acerca de consejería breve para reducir el consumo. Es decir, se pregunta al paciente cuánto alcohol consume y, si es un bebedor, se le da un consejo breve sobre cómo beber menos.

Marque su nivel de acuerdo con las siguientes afirmaciones (5 – Completamente de acuerdo, 4 – De acuerdo, 3 – Ni de acuerdo ni en desacuerdo, 2 – En desacuerdo, 1 – Completamente en desacuerdo

1. Los programas de tamizaje (detección) de consumo excesivo de alcohol que brindan consejos breves para beber menos son un método eficaz para reducir el consumo excesivo de alcohol.
2. Los centros de atención primaria o centros del primer nivel de atención en salud son un lugar apropiado para ofrecer programas de tamizaje de consumo excesivo de alcohol entre los pacientes y ofrecer consejos breves para beber menos

Marque los profesionales de salud que considere adecuados para realizar tamizaje y consejo breve para abordar el consumo excesivo de alcohol en atención primaria:

Médico/a

Enfermero/a

Psicólogo/a

Trabajador/a social

Obstetra / partera

Otro/a:

**2. Barreras**

A continuación, se presenta una lista de factores que pueden interferir en la práctica de la detección y consejo para reducir el consumo de alcohol excesivo. Lea cada ítem y marque hasta qué punto, en su opinión, **estos elementos representan una barrera** **en su país** **para implementar programas** de detección o tamizaje de consumo excesivo **de alcohol** y consejo breve para la reducción del consumo excesivo de alcohol (a partir de ahora “intervención breve”).

5 - Completamente de acuerdo - es una barrera muy importante

4 - De acuerdo – es una barrera

3 - Ni de acuerdo ni en desacuerdo

2 - En desacuerdo – no es una barrera

1 - Completamente en desacuerdo - no es una barrera para nada

* Cuando se menciona profesionales de salud, se refiere a aquellos que considere adecuados para la implementación.

1. El tamizaje e intervención breve para el consumo excesivo de alcohol es demasiado trabajo y requiere mucho esfuerzo
2. El tamizaje e intervención breve para el consumo excesivo de alcohol no son factibles en nuestra práctica clínica diaria en atención primaria
3. El tamizaje e intervención breve para el consumo excesivo de alcohol son culturalmente inapropiados
4. Los profesionales de la salud no tienen las habilidades para implementar tamizaje e intervención breve para el consumo excesivo de alcohol
5. Los profesionales de la salud creen que hacer tamizaje e intervención breve para el consumo excesivo de alcohol no ayudará a sus pacientes
6. Los profesionales de la salud consideran que el tamizaje e intervención breve para el consumo excesivo de alcohol no son su responsabilidad
7. Los profesionales de la salud creen que no pueden ayudar a los pacientes que consumen alcohol excesivamente
8. Los profesionales de la salud se muestran reacios a preguntar sobre el consumo excesivo de alcohol debido a las barreras sociales y culturales
9. Los profesionales de la salud carecen de tiempo suficiente para implementar tamizaje e intervención breve para el consumo excesivo de alcohol
10. La mayoría de los pacientes con consumo excesivo de alcohol piensan que su consumo de alcohol es normal, lo que dificulta la detección y el asesoramiento de los profesionales de salud
11. A los pacientes no les gusta hablar sobre su consumo de alcohol con su médico o enfermera
12. No hay ningún lugar para que los profesionales de salud refieran a pacientes con problemas graves de alcohol
13. No existen instrumentos para realizar tamizaje e intervención breve para el consumo excesivo de alcohol
14. Las pautas para realizar tamizaje y ofrecer intervención breve no son claras
15. Hay falta de incentivos financieros para ayudar a los profesionales de salud a realizar tamizaje y dar consejo breve para el consumo excesivo de alcohol
16. Hay falta de incentivos no-financieros para ayudar a los profesionales de salud a realizar tamizaje y dar consejo breve para el consumo excesivo de alcohol
17. Hay falta de apoyo continuo para ayudar a los profesionales de salud a realizar tamizaje e intervención breve para el consumo excesivo de alcohol
18. Los directores/jefes de los centros de atención primaria de salud no apoyan los programas de tamizaje e intervención breve para el consumo excesivo de alcohol
19. No hay cambios organizativos en los centros de atención primaria para la implementación de programas de tamizaje e intervención breve para el consumo excesivo de alcohol
20. No hay suficiente personal en los centros de atención primaria para la implementación de programas de tamizaje e intervención breve para el consumo excesivo de alcohol
21. Las leyes y regulaciones del país que determinan el precio y la disponibilidad del alcohol son demasiado indulgentes y liberales, lo que lleva a reducir las motivaciones de los profesionales de salud a realizar intervenciones breves para el consumo excesivo de alcohol
